# Unique genetic presentation of Gitelman syndrome in a Hispanic patient: Case report

**DOI:** 10.1177/2050313X251351547

**Published:** 2025-06-20

**Authors:** Aldo Arce, Matthew Nguyen, Dao Le, Ramy Hanna

**Affiliations:** 1 Division of Nephrology, Hypertension, and Transplant Nephrology, University of California, Irvine, CA, USA

**Keywords:** neurology

## Abstract

Gitelman’s syndrome, also known as, familial hypokalemia–hypomagnesemia, is a renal tubulopathy responsible for salt wasting resulting in, hypomagnesemia, hypocalciuria, and secondary activation of the renin–angiotensin–aldosterone system, responsible for the hypokalemia and metabolic alkalosis. Gitelman’s syndrome is due to a rare, autosomal recessive gene mutation due to deletions, missense, nonsense, frame-shift, and splice-site mutation in the solute carrier family 12, member 3 gene. Solute carrier family 12, member 3, encodes a thiazide-sensitive sodium–chloride cotransporter in the apical membrane of cells within the distal convoluted tubule, leading to the development of Gitelman’s syndrome. An 18-year-old male patient with a past medical history of chronic hypokalemia, hypomagnesemia, and constipation presented to the clinic as a continuation of care from his pediatric nephrologist for previously diagnosed Gitelman’s syndrome in his childhood. Regarding his history of Gitelman’s syndrome leading up to his diagnosis, at the age of 3, the patient was found to be hypokalemic on routine testing with no identifiable cause. The patient underwent genetic testing, where the test result demonstrated a positive solute carrier family 12, member 3 gene mutation, concurrent with Gitelman’s syndrome. On genetic testing, heterozygous variants were also detected c. 179C>T, which is a known disease-causing mutation. Currently, the patient is being monitored due to persistent hydronephrosis of the bilateral kidneys. The patient has constantly dealt with hydronephrosis; however, kidney function has been preserved. The c.179C>T (Thr60Met) variant has been used to identify individuals with Gitelman’s syndrome due to this mutation being the most common, thought to be found in 1–10/40,000 individuals, with higher prevalence within Asian demographics. Literature shows a greater association of Japanese and Chinese descent when compared to other demographic groups. This finding in an individual of Hispanic origin should increase suspicion that this finding can be found in individuals, not of Asian descent.

## Background

Gitelman’s syndrome (GS), also known as, familial hypokalemia–hypomagnesemia, is a renal tubulopathy responsible for salt wasting resulting in, hypomagnesemia, hypocalciuria, and secondary activation of the renin–angiotensin–aldosterone system, responsible for the hypokalemia and metabolic alkalosis.^1,2^ GS is due to a rare, autosomal recessive gene mutation due to deletions, missense, nonsense, frame-shift, and splice-site mutation in the solute carrier family 12, member 3 (*SLC12A3*) gene. SLC12A3 encodes a thiazide-sensitive sodium–chloride cotransporter (NCC) in the apical membrane of cells within a distal convoluted tubule (DCT), leading to the development of GS. Over 189 mutant alleles have been defined in individuals with known GS, causing abnormal protein processing and glycosylation.^3–5^

Mutations affecting the NCC result in decreased sodium reabsorption within the DCT. Increased sodium within the collecting ducts causes water loss, resulting in volume contraction due to loss of extracellular fluid. Reduced intravascular volume will cause activation of the renin–angiotensin–aldosterone system, increasing circulating levels of renin and aldosterone, respectively. Aldosterone increases the activity levels of the epithelial sodium channel (ENaC), which is in the cortical collecting duct, and absorbs sodium while increasing potassium excretion and hydrogen ion. ENaC activation results in hypokalemia and metabolic alkalosis, which is found in GS. Hypomagnesemia is also found in patients with GS. Magnesium channel, TRPM6 located within the DCT are less active due to absorption of Ca^2+^ within the proximal tubule leading to hypocalciuria, and hypomagnesemia present in GS.^2^ Reabsorption of Ca^2+^, by passive reabsorption within the proximal tubule and decreased Mg^2+^-channel TRPM6, located within the DCT. This explains the mechanism behind hypocalciuria and hypomagnesemia in GS.^2^

The prevalence of Gitelman syndrome is ~1–10/40,000, with higher prevalence being suspected in Asia.^2,6^ Individuals of Caucasian origin are also estimated to have a higher prevalence of GS, with ~1% of heterozygosity, making it one of the most common renal tubular disorders in this population.^2^

GS is usually detected in early adolescence or adulthood due to the condition being asymptomatic or associated with relatively mild, nonspecific symptoms such as muscular weakness, fatigue, salt craving, thirst, polyuria, constipation, or cramps.^6^ One serious medical problem that can occur is cardiac arrhythmia and even sudden cardiac death due to severe hypokalemia.^7,8^

We present an interesting case of Gitelman syndrome with the heterozygous variant c.179C>T, as well as the diagnostic workup and management of this patient.

## Case presentation

Written consent was obtained from the patient prior to this manuscript being written. This is an 18-year-old male patient with a past medical history of chronic hypokalemia, hypomagnesemia, and constipation who presented to the clinic as a continuation of care from his pediatric nephrologist for previously diagnosed GS in his childhood. Leading up to his diagnosis, at the age of 3 the patient was found to be hypokalemic on routine testing with no identifiable cause.

At that time, the patient underwent genetic testing, where the test result demonstrated a positive SLC12A3 gene mutation, concurrent with GS. On genetic testing, heterozygous variants were also detected c. 179C>T, which is a known disease-causing mutation, and c. 1387G>A, a variant of uncertain significance. As individuals with GS have an increased risk of arrhythmia, as ~50% of individuals have increased QT intervals, a routine electrocardiogram was performed.^2,6^ At 12 years old, the patient had ambulatory monitoring with a 24-h Holter cardiac monitoring, which showed no arrhythmias. Yearly electrocardiograms have also shown no abnormalities since the initial studies. The patient also struggles with chronic constipation because of hypokalemia and hypomagnesemia. The patient’s condition has been relatively stable apart from hypokalemia.

The only electrolyte that was consistently tracked for this patient was potassium. Initial tracking began when the patient was 3 years old due to a hypokalemic state being found on routine lab testing. Unfortunately, the initial lab value could not be retrieved due to this medical encounter not being electronically imported into the patient’s medical record. Since potassium monitoring began, the patient has remained within a range of 3.2–4.3 mmol/L range. Previous research suggests a potassium level of 3.0 mmol/L is ideal for patients with GS.^6^ Since consistent monitoring began in October 2016, the patient’s potassium was 3.2 mmol/L. Supplementation began with potassium chloride PO 80 mEq TID and a potassium-sparing diuretic were also added, amiloride 20 mg daily. This dose maintained patient potassium levels within normal limits until 2019, when potassium was 3.3 mmol/L. The patient had potassium supplementation increased to 100 mEq TID, and the amiloride dose was unchanged as he started puberty. The patient is currently on the following medication: potassium chloride 80 mEq TID and amiloride 20 mg daily. These medications are adjusted as needed to control potassium troughs that can occur in GS patients. However, the patient has maintained potassium levels of 3.6–4 mmol/L without further adjustment of medications. Other medications taken by the patient are sodium chloride 1000 mg BID, dicyclomine 100 mg/5 mL, simethicone 80 mg BID, lactulose 10 g/15 mL, Miralax 17 g, and cholecalciferol 1000 IU. Supplementation with sodium chloride is used to counteract the excessive salt wasting, which is the hallmark of GS. Patients also tend to lose the chloride ion more, which is another reason sodium chloride is used, as well as potassium chloride supplementation for this patient. Furthermore, cholecalciferol supplementation is given as the patient has been found to be vitamin D deficient, possibly secondary to GS or due to limited sun exposure or insufficient dietary intake.

When the patient was assessed at the clinic during his transition from pediatric nephrology to an adult nephrologist, he was found to have no symptoms associated with GS except for chronic constipation. The patient did not have heart palpitations, headache, dizziness, numbness or tingling of extremities, joint pain or stiffness, or anemia. His creatinine was 0.63 mg/dL, potassium 3.9 mmol/L, magnesium 1.8 mg/dL, calcium 9.9 mg/dL, hemoglobin 14.7 g/dL, and bicarbonate 29 mmol/L. As previously mentioned, the patient undergoes yearly cardiac monitoring, which shows no arrhythmias. Currently, the patient is being monitored due to persistent bilateral mild hydronephrosis. Initially, the patient was diagnosed with mild left hydronephrosis at the age of 3 years old. A voiding cystourethrogram was performed at this time, which was negative. At 13 years old, a renal ultrasound was performed in November of 2021 and found isolated improvement of the left hydronephrosis with no evidence of obstruction. However, an interval ultrasound in March 2022, found persistent bilateral hydronephrosis with preserved renal function as noted by an estimated glomerular filtration rate (eGFR) of ~95. In July 2022, the patient underwent reevaluation for bilateral hydronephrosis through uroflow analysis, which revealed an unobstructed urination without retention. An ultrasound was also performed at this time, showing left grade 2 hydronephrosis and right mild pelviectasis. Since these visits, the patient has had resolution of right hydronephrosis, with mild persistent hydronephrosis of the left kidney. He is being monitored through routine lab work to assess through eGFR and yearly renal ultrasounds to monitor bilateral kidney status. Unfortunately, imaging results are not available as the patient has not undergone imaging since being brought into our care. The patient is followed with in-person every 4 months to assess treatment efficacy in preventing episodes of hypokalemia and hypomagnesemia, which had occurred in the past.

## Discussion

As previously mentioned, GS is thought to be found in 1–10/40,000 individuals with a higher prevalence within Asian demographics.^3,6^ Caucasian individuals also have a high prevalence, with an estimated 1% being heterozygous for common renal tubular disorders.^2^ The patient we present is different, as he is of Hispanic descent, which has not been found to be strongly associated with GS, making this a unique case, further expanding the originality of this case.

The c.179C>T (Thr60Met) variant has been used to identify individuals with GS due to this mutation being the most common, even though more than 500 mutations have been identified in the SLC12A3 gene.^9^ This mutation is due to a C to T substitution at base 179 within exon 1, at codon 60.^10^ This finding in the literature shows greater association with individuals of Asian descent, particularly Japanese and Chinese, when compared to other demographic groups.^11–14^ This finding in an individual of Hispanic origin should increase suspicion that this finding can be found in individuals not of Asian descent. Furthermore, research has also found the c.179C>T variant to be associated with autoimmune thyroiditis, which can impact the care of this patient as this is an extra-renal manifestation.^15^ Clinicians with suspicion of GS should have genetic testing performed on patients who they believe have a high likelihood of being GS positive.

Laboratory findings show significant electrolyte abnormalities, and it is one of the initial presentations in patients with GS. As previously mentioned, salt loss leading to hypokalemia, hypomagnesemia, or a combination of the two suggest further workup. Additional findings that help support the diagnosis of GS are chronic hypokalemia <3.5 mmol/L with inappropriate renal potassium wasting, metabolic alkalosis, hypomagnesemia <0.7 mmol/L, hypocalciuria, elevated renin levels or activity, increased fractional excretion of chloride >0.5%, low or normal blood pressures, and normal renal ultrasound.^6^ Currently, the only established diagnosis for the identification of GS is the use of genetic testing for SLC12A3 biallelic inactivation mutations (sensitivity of 90%–100% and specificity of 100%).^3^ For patients who do not have two mutations within SLC12A3, the sensitivity decreased from 80% to 65% depending on the genetic test performed. Management of GS is through supplementation of potassium, sodium chloride, and magnesium as needed. The dosage of magnesium supplementation should be adjusted to decrease the risk of diarrhea.^3,6^

Evidence of hypokalemia in patients with GS should prompt routine electrolyte monitoring to prevent arrhythmias and pseudogout due to known electrolyte imbalances. Pseudogout has also been reported in patients with GS due to hypercalcemia, resulting in calcium pyrophosphate dehydrated crystals being deposited in the synovium and synovial fluid. Pseudogout attacks are treated with magnesium supplementation and non-steroidal anti-inflammatory drugs.^2^

Even though the symptoms stated above are conditions that are seen in patients with GS our patient has not displayed them. The patient was found to be hypokalemic on routine lab testing as a 3-year-old child. Since his initial presentation, our patient has been screened for cardiac arrhythmia with electrocardiograms, which have shown no abnormalities. Furthermore, the patient has been treated with a high-sodium and high-potassium diet after diagnosis. Oral potassium chloride, sodium chloride, and amiloride have been prescribed to manage and prevent electrolyte abnormalities in this patient. Adequate hydration and nutrition of potassium-rich food have also been provided to minimize electrolyte abnormalities.

Limitations within this case report are due to a lack of access to the initial presenting condition of the patient during the first admission. There was also a lack of systematic lab electrolyte monitoring within the electronic medical records. This limited some of the electrolytes that are beneficial in examining patients with GS, such as creatinine, magnesium, and calcium. Medical records would reference hypomagnesemia without supporting lab values. Imaging results related to bilateral hydronephrosis were also documented, but measurements were not listed. The final limitation is, as inherent in all case reports, that the information and case provided do not seek to provide a cause-or-effect relationship and are not able to be generalizable to a larger population.

## Conclusion

In conclusion, GS is a rare but significant renal tubulopathy that is characterized by salt wasting and a combination of electrolyte imbalances, including hypokalemia, hypomagnesemia, and hypocalciuria. The genetic mutation in the SLC12A3 gene impairs sodium reabsorption in the DCT, triggering a cascade of events that lead to electrolyte abnormalities. In this patient, the genetic analysis identified the c.179C>T mutation, a common mutation linked to GS, although its presence in a Hispanic patient suggests that clinicians should consider a broader ethnic range when diagnosing GS.

This patient’s clinical course has been relatively stable, with consistent management of his electrolyte imbalances through potassium and magnesium supplementation. Despite challenges such as chronic constipation and the potential for renal complications like hydronephrosis, the patient’s careful monitoring and appropriate treatment have mitigated the major risks associated with GS. The case also emphasizes the importance of regular follow-up, including cardiac monitoring and laboratory tests, to prevent life-threatening complications. Although the limitations of the case, such as incomplete early medical records, affect the overall analysis, the patient’s ongoing management exemplifies a personalized and proactive approach to treating GS. This case reinforces the importance of genetic testing and tailored treatment plans in managing rare disorders such as GS.
